# Brain Age Prediction Using Multi-Hop Graph Attention Combined with Convolutional Neural Network

**DOI:** 10.3390/bioengineering11030265

**Published:** 2024-03-08

**Authors:** Heejoo Lim, Yoonji Joo, Eunji Ha, Yumi Song, Sujung Yoon, Taehoon Shin

**Affiliations:** 1 Division of Mechanical and Biomedical Engineering, Ewha W. University, Seoul 03760, Republic of Korea; dobby923@naver.com; 2 Graduate Program in Smart Factory, Ewha W. University, Seoul 03760, Republic of Korea; 3 Ewha Brain Institute, Ewha W. University, Seoul 03760, Republic of Korea; yoonjijoo@ewha.ac.kr (Y.J.); youme.a.song@gmail.com (Y.S.); 4 Department of Brain and Cognitive Sciences, Ewha W. University, Seoul 03760, Republic of Korea

**Keywords:** brain age prediction, brain magnetic resonance image, graph attention, self-attention, convolutional neural network

## Abstract

Convolutional neural networks (CNNs) have been used widely to predict biological brain age based on brain magnetic resonance (MR) images. However, CNNs focus mainly on spatially local features and their aggregates and barely on the connective information between distant regions. To overcome this issue, we propose a novel multi-hop graph attention (MGA) module that exploits both the local and global connections of image features when combined with CNNs. After insertion between convolutional layers, MGA first converts the convolution-derived feature map into graph-structured data by using patch embedding and embedding-distance-based scoring. Multi-hop connections between the graph nodes are modeled by using the Markov chain process. After performing multi-hop graph attention, MGA re-converts the graph into an updated feature map and transfers it to the next convolutional layer. We combined the MGA module with sSE (spatial squeeze and excitation)-ResNet18 for our final prediction model (MGA-sSE-ResNet18) and performed various hyperparameter evaluations to identify the optimal parameter combinations. With 2788 three-dimensional T1-weighted MR images of healthy subjects, we verified the effectiveness of MGA-sSE-ResNet18 with comparisons to four established, general-purpose CNNs and two representative brain age prediction models. The proposed model yielded an optimal performance with a mean absolute error of 2.822 years and Pearson’s correlation coefficient (PCC) of 0.968, demonstrating the potential of the MGA module to improve the accuracy of brain age prediction.

## 1. Introduction

The rates of brain aging are heterogeneous across individuals, affected by various genetic, environmental, and lifestyle conditions [1,2] The resultant biological brain age (or simply brain age) can be predicted by applying machine learning algorithms to neuroimaging data, which are assumed to reflect aging-related changes in brain tissues [3,4,5,6,7]. The difference between the predicted biological brain age and chronological age represents the brain age gap or brain age delta, which is considered an index of the deviation from a normal aging trajectory [8,9]. A significant association exists between brain neuroimaging data and neurodegenerative disorders [10], and many studies demonstrated that the positive brain age gap, which is extracted from brain MRI, can serve as a biomarker for degenerative neurological disorders, such as dementia [11,12], Alzheimer’s disease [13,14], schizophrenia [15,16], traumatic brain injuries, chronic pain, and others [17,18].

Machine learning regression models need to be trained by using the brain images of healthy subjects whose expected brain ages equate to their chronological ages. Early approaches manually extracted anatomical features from brain magnetic resonance imaging (MRI), such as cortical thickness and regional or tissue-specific volumes, and fed them into traditional regression models, such as linear regression [19], support vector regression (SVR) [20], and Gaussian process regression (GPR) [3]. Gaun et al. predicted brain age by partial least squares regression (PLSR) by using cortical thickness features and yielded an MAE of 7.90 years [21]. Raw image pre-processing for feature extraction involves multiple steps such as field-offset correction, removal of the non-brain region, and tissue segmentation, which may be too time consuming for clinical practice. Furthermore, the pre-engineered features may be suboptimal as they were chosen based on generic information about the brain, not specific relevance to brain age.

Deep artificial neural networks (a.k.a. deep learning) have been used ubiquitously in medical image analysis, encouraged by the remarkable success of convolution neural networks (CNNs) in computer vision applications [22]. A CNN iteratively updates the convolution-filtered features of raw or minimally processed input images in a way that reduces a pre-defined prediction error metric. This ensures the identification of the set of features directly suited for increasing prediction accuracy without the need for time-consuming pre-processing steps. Since the first CNN application to brain age prediction by Cole et al. [23], established CNN architectures verified on natural images or their variants have emerged, including VGG, ResNet, and DenseNet [5,24,25]. More advanced CNN architectures have since been developed to boost the representational power and improve prediction accuracy. Lam et al. [26] employed attention modules between intermediate and final convolution layers such that the relationship between the feature maps of different scales is learned. Cheng et al. [27] proposed two-stage network architectures with novel ranking losses, where the first network estimated discretized coarse ages that were further fine-tuned to continuous ages through the second network. He et al. [28] split MR images into two image channels representing contrast and morphometry information and used an attention mechanism for the optimal fusion of the two channels. Zhang et al. [29] proposed the Anatomical Feature Attention enhanced 3D-CNN (AFAC) by using both the anatomical and deep features of brain sMRI and yielded an MAE of 2.20 years. While the source, sample size, and age range of the training and test data varied significantly across the studies, the resultant mean absolute errors (MAEs) ranged from 2.14 years to 5.55 years, adequately supporting the technical feasibility of CNN-based brain age prediction.

CNNs focus mainly on spatially local features since small-size convolutional filters are learned to find pixel relationships in a local neighborhood. Though more global feature characteristics can be learned by repeating the convolutional layer followed by a pooling layer, a CNN alone cannot efficiently capture connective information between distant regions. This limitation may be critical particularly for brain age prediction since different brain regions interact with one another through their neurological connections in an orchestrated manner rather than in isolation [30]. To address this issue, deep learning models inspired by self-attention mechanisms have been developed (e.g., Transformers). Jun et al. [31] proposed a convolutional encoder for high-level feature extraction from two-dimensional (2D) image sets and a Transformer that captures the dependencies of distant slices via an attention mechanism (MAE = 3.49 years; age range = 5.82 to 75.29 years). He et al. [32] proposed a global and local Transformer that fused the fine-grained features obtained from image patches and the global context features obtained from the entire image (MAE = 2.70 years; age range = 0 to 97 years). 

A graph neural network (GNN) is another promising framework for utilizing the inter-region relationship due to its suitability for graph-structured data represented by nodes and their connections (edges). Kawahara et al. [33] proposed a GNN architecture that processes diffusion-MRI-derived brain connectivity data while considering brain network topological locality. Liu et al. applied graph convolutional neural networks to cortical surface mesh data to predict brain age for pre-term neonates [34]. These methods require graph structures of data such as tractography networks [33] or surface meshes [34], which must be generated from raw MR images. Cai et al. proposed a graph Transformer framework to fully utilize multi-modal MR images including T1 and diffusion tensor images. While being capable of learning regional and inter-modality relationships, this approach needed the registration of multi-modal images based on the standard brain template [35].

In this work, we aim to develop neural network architectures that exploit both local and global features directly from minimally processed raw images to improve the accuracy of brain age prediction. To this end, we propose a novel multi-hop graph attention (MGA) module that can be easily plugged into and complement existing CNNs to learn local features and their inherent relationships. Since the input data type is an image, the MGA module converts the image features extracted by the preceding convolutional layers into graph nodes through patch splitting and embedding. The graph edges are initialized to represent the distance score between the patch embeddings and updated in a fashion that considers indirectly connected nodes across multi-hops based on Markov chain modeling. That is, the inter-regional connectivity is learned during training rather than pre-defined as in GNNs by using DTI-derived graph data. The final architecture is formed by combining the MGA module with sSE (spatial squeeze and excitation)-ResNet18 [36] and optimizing the MGA hyperparameters. The proposed MGA-sSE-ResNet18 is compared with established, general-purpose CNNs as well as recent deep learning models specialized for brain age prediction.

## 2. Materials and Methods

### 2.1. Overview of Multi-Hop Graph Attention

**Figure 1:**
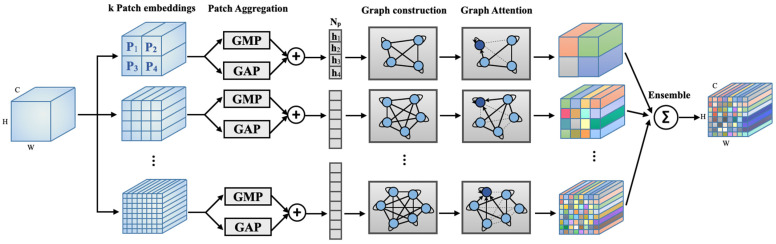
An overview of the proposed multi-hop graph attention (MGA) module. MGA contains *k* independent branches that handle patch embeddings of different sizes. Each branch constructs a node set by using patch embedding and aggregation, as well as an edge set based on similarity scores among the node embeddings. Graph attention is applied to update the node set with consideration of multi-hop inter-node relationships. The updated feature patches from different branches are ensembled to obtain a final MGA output.

A schematic diagram of the proposed MGA module appears in Figure 1. Placed between convolution layers, the module receives a feature map extracted from the preceding convolution layer and yields an updated feature map to the following layer. The updated feature map’s size remains identical to the input feature map such that a skip connection can be used across the module. The MGA module first constructs graph sets by defining nodes through patch embedding and aggregation and edges through inter-node similarity calculations. The formed graph sets pass through a graph attention block that updates the patch set based on multi-hop self-attention and obtains an updated feature map. This procedure repeats for multiple sets of patches of different sizes, and the resulting feature updates are combined to produce the module’s final output.

### 2.2. Graph Construction

We obtain Np patches from the feature map of size C × H × W × D obtained from the previous convolution layer. A patch size of C × H//*ϒ* × W//*ϒ* × D//*ϒ* and a stride of min(H//*ϒ*, W//*ϒ*, D//*ϒ*) are used where *ϒ* is a hyperparameter. The set of patches is denoted as **P** = {**p**_1_, **p**_2_, …, **p**_Np_}, **P** ∈ ℝ^NpxS^, where S is the vectorized size of a single patch. The parcellation occurs only along the three spatial dimensions to focus on the relationships among the spatial regions. Since the MGA module is applied after every convolution layer, which progressively decreases in spatial resolution, the patch size decreases as the entire network goes deeper. We aggregate each patch’s spatial channel dimensions by using global average pooling (GAP) and global max pooling (GMP). The two pooled tensors are then concatenated such that the resultant tensor **H**^Npx2^ contains the highlighted feature description of the patch set. This can be represented as **H** = {**h**_1_, **h**_2_, …, **h**_Np_}, **h**_i_ ∈ ℝ^2^ and serves as a set of nodes for graph construction.

The size of a patch corresponding to each node is determined by the hyperparameter *ϒ*. With a larger *ϒ*, more local patches will be embedded in a larger number of nodes and the relationship will be explored by the subsequent graph attention block. We consider multiple patch embeddings of varying sizes to offer rich representations of the input feature map. The multiple patch sets will be processed in parallel and ensembled at the end of the MGA module.

An edge is another component composing a graph and represents the connective strength between local anatomical features in the present study. We define the edge *e_ij_* between two nodes **h**_i_ and **h**_j_ by computing a Euclidean-distance-based similarity score of two feature embeddings **Vh**_i_ and **Vh**_j_ as follows:

(1) $$e_{ij}\;:=1/(exp({\left\|Vh_{i}-Vh_{j}\right\|}_{2}))$$

The use of the learnable embedding **V** (∈ℝ^2×2^) is supported by the fact that the brain region connections are viewed based on a functional and effective linking of neural elements rather than the direct similarity of the image features [30]. The graph based on this edge definition is undirected since *e_ij_* = *e_ji_* for all *i*, *j* < N_p_, and therefore, the corresponding edge matrix, denoted as **E**, is symmetric. It is noteworthy that the similarity score between the same node embedding is 1, implying that every node can receive attention not only from its neighbors but also itself.

### 2.3. The Multi-Hop Neighborhood of Nodes

**Figure 2:**
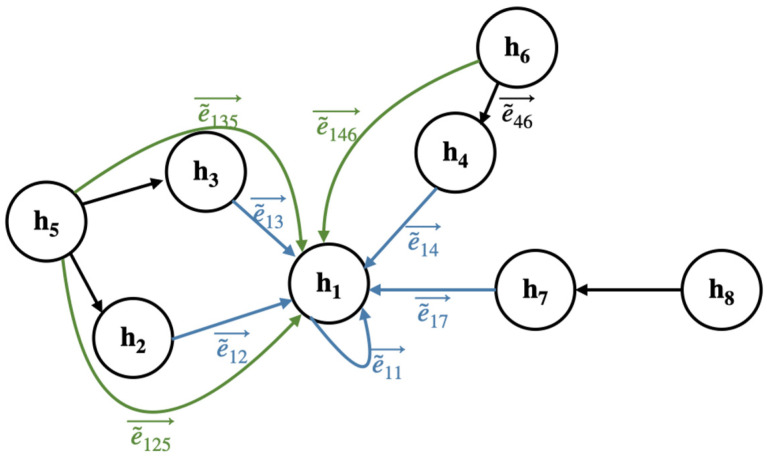
Illustration of the dynamics of multi-hop transitions across nodes in the graph structure.

In this section, we modify the edge matrix **E** to consider both the direct and indirect neighborhood of the node set through a statistical model of multi-hop connections among the nodes. We consider a random event of moving from one node to another in the state space defined as **Ω** = {**h**_1_, **h**_2_, …, **h**_Np_}. The transition may simply occur between two nodes, but it can also occur sequentially in three or more nodes (e.g., *ẽ*_46_, *ẽ*_14_, and *ẽ*_11_ in Figure 2). Denoting the outcome of the *t*-th transition by X(*t*) with time step *t* ∈ T = {0, 1, 2, …, *m*} (*m* ∈ ℝ), we model the similarity score (i.e., edge) as the conditional probability of transitioning from state **h**_j_ to **h**_i_, expressed as P(X(*t*) = **h**_i_|X(*t* − 1) = **h**_j_). To equate the summation of all the edges entering a specific node to 1, the edge matrix is row-wise normalized to **Ẽ** = **D**^−1^**E**, where **D** is a diagonal matrix with diagonal element d_ii_ = ∑_j_ e_ij_. The normalized edge matrix **Ẽ** is stochastic, satisfying the following equations for all *i*, *j* = {0, …, N_p_}:

(2) $$\left\{\begin{array}{c} 1\geq {\tilde{e}}_{ij}\geq 0\\ \sum_{j=0}^{N_{p}}{\tilde{e}}_{ij}=1 \end{array}\right.$$

Typical implementations of graph attention networks (GATs) consider the influence of a node in the first-order domain adjacent to it when integrating a graph structure [37,38]. To enhance the representational capacity of the graph network, we modify the edge matrix such that it considers up to m^th^-order neighborhood information.

Consider an m-hop transition probability that a process currently in state **h**_j_ transitions to state **h**_i_ through *m* (>=1) hops, denoted by *ẽ_ij_*^(*m*)^. We assume that the transition event satisfies the key Markov property, i.e., depends only on the most recent state. Then, *ẽ_ij_*^(*m*)^ can be expressed as below by using the Chapman–Kolmogorov equation:

(3) $${\tilde{e}}_{ij}^{\left(m\right)}=P\left(X\left(m\right)=h_{i}|X\left(0\right)=h_{j}\right)\;=\sum_{k=0}^{N_{p}}P\left(X(m)=h_{i},\;X(m-1)=h_{k}|X(0)=h_{j}\right)\;=\sum_{k=0}^{N_{p}}P\left(X(m)=h_{i}|X(m-1)=h_{k}\right)\cdot P\left(X(m-1)=h_{k}|X(0)=h_{j}\right)\;=\sum_{k=0}^{N_{p}}{\tilde{e}}_{ik}\cdot {\tilde{e}}_{kj}^{\left(m-1\right)}$$

where ẽ_ij_^(0)^ is set to *ẽ_ij_* for initialization. Equation (3) indicates that the probability of m-hop transitions occurring equals the multiplication of the products of all the intermediate transitions recursively. In matrix form, this can be written as follows:

(4) $${\tilde{E}}^{\left(m\right)}=\tilde{E}\;{\tilde{E}}^{\left(m-1\right)}={\tilde{E}}^{\left(2\right)}\;{\tilde{E}}^{\left(m-2\right)}=\cdots ={\tilde{E}}^{m}$$

Accounting for zero-hop up to *m*-hop transitions, we obtain the *m*-th order edge matrix, denoted as ${\tilde{E}}_{\forall }^{\left(m\right)}$, as follows:

(5) $${\tilde{E}}_{\forall }^{\left(m\right)}=\tilde{E}+\beta {\tilde{E}}^{\left(2\right)}+\beta^{2}{\tilde{E}}^{\left(3\right)}+\cdots +\beta^{m-1}{\tilde{E}}^{\left(m\right)}=\sum_{k=1}^{m}\beta^{k-1}{\tilde{E}}^{k}$$

where *β* is a hyperparameter ranging from 0 to 1, which increases the weight on edge matrices for smaller hops.

${\tilde{E}}_{\forall }^{\left(m\right)}$ is no longer symmetric, which violates the requirement of the undirected graph that ${\tilde{e}}_{\forall ,ij}^{\left(m\right)}$ should be equal to ${\tilde{e}}_{\forall ,ji}^{\left(m\right)}$. Therefore, the final multi-hop edge matrix is defined as a summation of ${\tilde{E}}_{\forall }^{\left(m\right)}$ and ${\tilde{E}}_{\forall }^{\left(m\right)T}$ in Equation (6):

(6) $$E_{\forall }^{\left(m\right)}:=({\tilde{E}}_{\forall }^{\left(m\right)}+{\tilde{E}}_{\forall }^{\left(m\right)T})/2$$

Note that a larger *m* allows for more global relationships to be considered in the node-set **H**, but this may undermine the relative importance of local relationships. This tradeoff will be investigated by experiments with varying *m* values (Section 4.1).

### 2.4. Updating Nodes via Graph Attention

We update the node set by adapting the masked graph self-attention proposed by Velickovic et al. [39]. The attention coefficient ɑ_ij_, which quantifies the relevance of node **h**_j_ to node **h**_i_, can be expressed as

(7) $$\alpha_{ij}=\frac{exp\left(LeakyReLU\left(W^{T}\left[Vh_{i}∥Vh_{j}\right]\right)\right)}{\sum_{k\in N_{i}}exp(LeakyReLU\left(W^{T}\left[Vh_{i}∥Vh_{k}\right]\right)}$$

where || is the concatenation operation, **W** is a learnable weight matrix (∈ℝ^4×2^), and *N_i_* is the subset of **H** that is related to the target node **h**_i_. In conventional graph networks, all edges e_ij_ are binary, representing a direct connection (one) or disconnection (zero) between nodes **h**_i_ and **h**_j_ [40,41,42]. The masked self-attention (within the subset *N_i_*) can be implemented by computing **W**[**Vh**_i_ || **Vh**_j_] only for **h**_i_ and **h**_j_ whose edge values are 1 s. In our work, edges have continuous values due to the use of distance-based scores (Equation (1)) and are therefore made binary by using a threshold *θ*:

(8) $$E_{\forall }^{\left(m\right)}=\left\{\begin{array}{c} 1,\;if\;E_{\forall }^{\left(m\right)}>\theta \;\\ \;0,\;otherwise \end{array}\right.$$

where *θ* is empirically chosen as the average of ${\tilde{E}}_{\forall i,i+1}$ over i = {1, 2, …, *N_p_*−1}. This value represents the average edge strength between directly connected nodes, which is the only connection considered in conventional GNNs. The updated output patch set **P**′^Np×S^ is obtained thereafter by an attention-weighted linear transformation of the input patch set **P**^Np×S^:

(9) $${p\prime }_{i}=\sum_{j\in N_{i}}\left(\alpha_{ij}\cdot p_{j}\right)$$

The set of updated local patches is combined to form an updated whole feature map of size C × H × W × D. Recall that *k* updated feature maps will be available through the parallel processing of *k* patch sets obtained by using different *ϒ*. For each of the parallel processing, different patch-embedding matrices **V** (in Equation (1)) and weight matrices **W** (in Equation (7)) are used. The resultant *k* feature maps are averaged and passed over to the following convolution layer.

### 2.5. MGA-sSE-ResNet18

**Figure 3:**
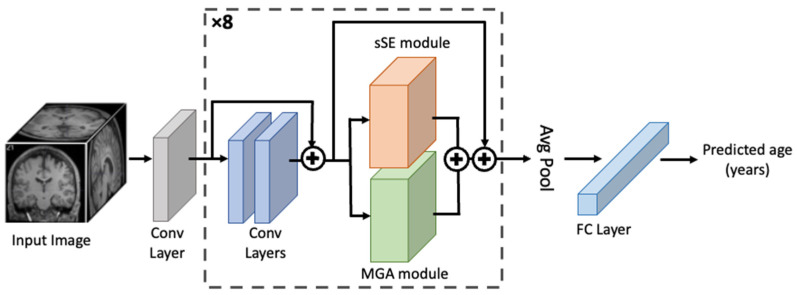
Overview of the proposed MGA-sSE-ResNet18 for brain age prediction. The dotted box, a key network component, consists of two convolutional layers followed by a parallel combination of the sSE and MGA modules and is repeated eight times. Residual connection applies across the two convolutional layers and across the combination of the sSE and MGA modules.

Figure 3 presents the overall architecture of the proposed model for brain age prediction. While the MGA module can be incorporated flexibly into any CNN, we choose ResNet18 combined with the spatial squeeze-and-excitation (sSE) module, hereafter termed sSE-ResNet18 as a backbone model. sSE-ResNet18 has been well validated in numerous prediction tasks including brain age prediction [43,44]. The model starts with a convolutional layer (the grey box in Figure 3) and consists of eight consecutive blocks (the dotted box) followed by a pooling layer and one FC layer for regression. Each block contains two convolutional layers, followed by one sSE module (orange) combined in parallel with one MGA module (green). The outputs of the sSE and MGA modules are combined through averaging. Skip connections are used across the parallel combination of sSE and MGA modules as well as the two convolutional layers.

The number of transition hops *m*, the patch split ratio *ϒ*, the number of branches *k*, and the multi-hop weight coefficient *β* are key hyperparameters of the proposed model, determined through iterative search. The effect of *m* was first examined in the range from 1 to 8 while *ϒ* = 2 and 4 were used for two branches (*k* = 2) with a multi-hop weight coefficient *β* = 1. Different combinations of the patch split ratio and number of branches were examined subsequently, including *k* = 1 with *ϒ* = 2, 4, or 6; *k* = 2 with *ϒ* = 2 for both branches; *k* = 2 with *ϒ* = 2 and 4 for two branches; and *k* = 2 with *ϒ* = 2 and 6, while *m* and *β* were set to 3 and 1.0, respectively. After fine-tuning *m*, *ϒ*, and *k*, the effect of *β* was last examined from 0.7 to 1.0 at the interval of 0.05. The detailed information for MGA-sSE-ResNet18 is outlined in Table 1, where the hyperparameter values for the MGA module are based on the fine-tuning results shown in Section 4.1.

## 3. Experiments

### 3.1. Dataset

**Figure 4:**
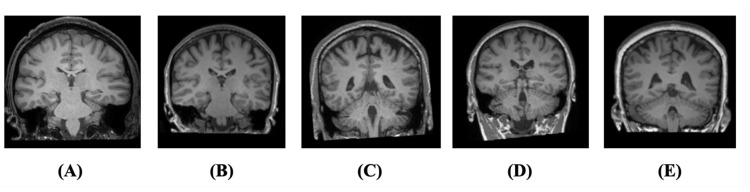
Sample images of each age group of T1 brain MRI: (**A**) aged 20, (**B**) aged 30, (**C**) aged 40, (**D**) aged 50, and (**E**) aged 60.

Three-dimensional whole brain T1-weighted MR images of 2788 healthy subjects were obtained from seven public datasets: OpenNeuro [45], COBRE [46], Open fMRI [47], INDI [48], IXI [49], FCP1000 [50], and XNAT [51]. The demographic information and example images of the datasets are shown in Table 2 and Figure 4, respectively. The MRI subjects were aged between 20 and 70 years with a mean of 37.53 years and a standard deviation of 16.50 years. There were 1337 males and 1451 females among the subjects, with the two sex groups having similar age distributions with *p* < 0.001. Minimal pre-processing was performed on the collected brain MR images by using the Statistical Parametric Mapping version 12 (SPM12) software package (University College London, UK) running in MATLAB version R2017b (the MathWorks Inc., Natick, MA, USA). Specifically, MR images were resampled to an isotropic voxel size of 1.2 mm × 1.2 mm × 1.2 mm with a matrix size of 101 × 101 × 121. The histogram equalization and N4 bias field correction [52] were performed to harmonize the MR images from multiple sources. All the images were scaled by using min–max normalization.

### 3.2. Experimental Settings

We randomly divided the age-labeled brain MR images into three subsets with similar age distributions: (1) the training dataset (70%, 1951 samples), (2) the validation dataset (15%, 419 samples), and (3) the test dataset (15%, 418 samples). Data augmentation was performed by using random rotation and translation during training. The rotation angle was between −20° and 20°, and the translation distance was within [–6, 6] voxels for each direction with uniform probability. The primary training parameters were batch size = 12, an Adam optimizer with an initial learning rate of 0.005, and a weight decay of 0.0001. The learning rate decreased by 0.1 every 70 epochs to stabilize the convergence. The hybrid loss function based on Spearman’s rank correlation coefficient and traditional mean squared error (MSE) was used as proposed by Cheng et al. [27]. The drop path was applied to each MGA branch to mitigate overfitting and accelerate the learning process by reducing the model parameters [53]. To compensate for the systemic bias toward the mean age of the training dataset, a linear age-bias correction procedure was performed as suggested in [54]. The final network was chosen based on the performance of the validation dataset. Each method’s predictive performance was evaluated by calculating the mean absolute error (MAE) and Pearson correlation coefficient (PCC) on the test dataset. All the model training and testing were implemented with two NVIDIA A6000 GPUs (total memory of 96 GB).

### 3.3. Comparisons with Other Models

The proposed MGA-sSE-ResNet18 was first compared with the same backbone, but the MGA module was replaced with multi-head self-attention (MSA-sSE-ResNet18), the key component in attention-based architecture such as Transformers and Vision Transformers (ViTs) [55,56]. Both MGA and MSA employ query-key-based self-attention between patch embeddings but differ in the scope of the key vectors to attend to and the type of alignment functions. MSA was applied after the patch embedding combined with positional encoding as in ViTs. MSA was implemented by using a patch size of five, an embedding size of 512 per patch, and six attention heads.

We compared the proposed model with popular CNNs for natural image recognition. The compared network architectures included (1) ResNet18 (the most established CNN model), (2) sSE-ResNet18 (ResNet18 incorporating channel squeeze and a spatial excitation block), (3) DenseNet121 (as an example of a CNN model with a large number of parameters) [57], and (4) MoblieNetV2 (as an example of lightweight models) [58]. We also performed comparisons with novel CNN-based architectures specifically designed for brain age estimation, including (5) simple fully convolutional networks (SFCNs) [8] and (6) two-stage age networks (TSANs) by using the ranking loss [27]. These two methods were chosen as they yielded superior performance by using 3D CNN-based architectures with the input of the 3D T1-weighted MR images only as in this study. The SFCN optimized a lightweight CNN model and showed a state-of-the-art performance when using a large training dataset (n = 12,949). The TSAN predicted the rough brain ages at the first stage and then fine-tuned the prediction with the discretized brain age as an additional input. The learning environment of all the compared models was set to the same as described in Section 3.2, except for the SFCN and TSAN, which used the hyperparameters that were used in the original papers.

The proposed MGA module was incorporated into two backbone networks, ResNet18 and sSE-ResNet18, for comparisons. We investigated the sex label’s effect as an additional input to our final model MGA-sSE-ResNet18, inspired by its similar use in the TSAN. We converted the sex label into a two-dimensional one-hot encoding vector and concatenated it with the feature vectors extracted from the last block of convolutional layers and MGA modules (i.e., immediately prior to the fully connected layers).

## 4. Results

### 4.1. Hyperparameter Evaluation in MGA-sSE-ResNet18

**Figure 5:**
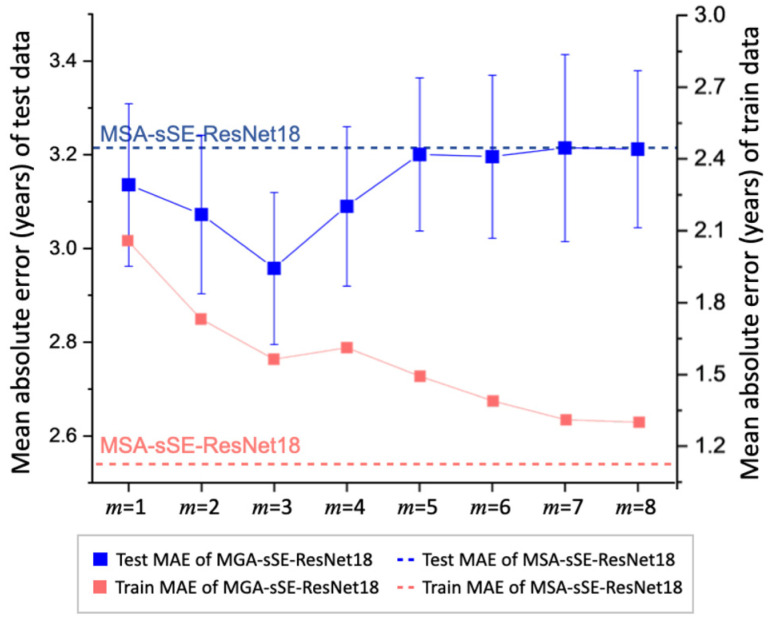
Performance of MGA-sSE-ResNet18 with different hop sizes (*m*) for brain age prediction. The test MAEs are represented in blue with the scale on the left vertical axis, and the training MAEs are represented in red with the scale on the right axis. The errors of MSA-sSE-ResNet18 are depicted as dotted lines, with blue and red representing the MAEs of test and training datasets, respectively.

Figure 5 displays the effect of the hop size on the performance of the proposed MGA-sSE-ResNet18. The MAE on the test dataset (in solid blue) mirrors a V-shaped curve for hop sizes *m* = 1 through 5 and saturates for *m* larger than 5. This implies that incorporating indirect connections across the nodes in the MGA module improves the representational power. Yet, considering the relationships of too many nodes may lead to worse generalizations. Overfitting induced by a large *m* is also confirmed by the training MAE (in solid red), which decreases over *m* nearly monotonically and becomes very low when *m* > 5. The optimal performance is achieved with *m* = 3, which results in the minimal MAE of 2.957 years and maximal PCC of 0.9584.

**Figure 6:**
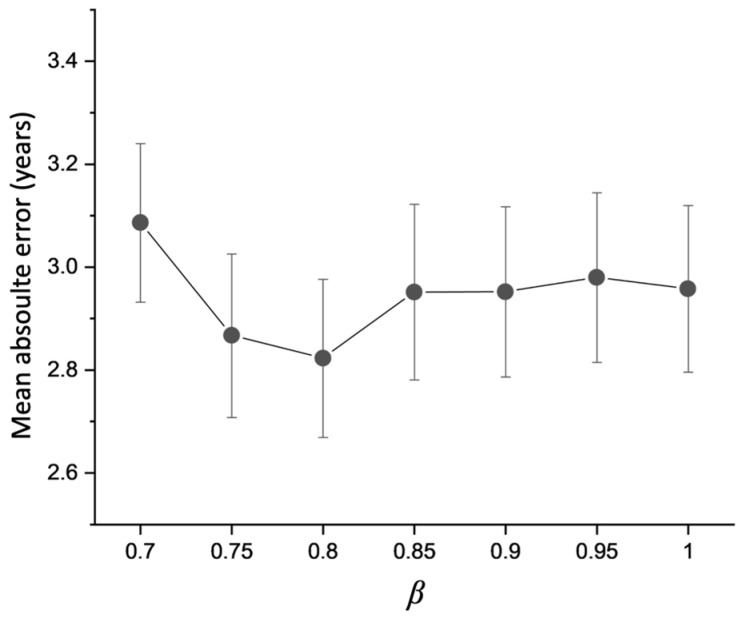
Effect of different *ϒ* and *k* combinations on brain age prediction of MGA-sSE-ResNet18. The prediction errors obtained using one branch (*k* = 1) or two branches (*k* = 2) are colored in gray and red, respectively.

Figure 6 illustrates the effect of different patch combinations determined by the number of attention branches *k* and patch split ratio *ϒ*. Due to a GPU memory limitation, only up to two branches (*k* = 2) were tested while *ϒ* varied from two to six. When a single patch split ratio was used (*k* = 1), *ϒ* = 4 presented a lower MAE value than *ϒ* = 2 and *ϒ* = 6. When two patch split ratios were used through two branches (*k* = 2), *ϒ* = {2,4} yielded a lower MAE than the two other combinations (*ϒ* = {2,2}, {2,6}) and all cases of a single branch. This implies that MGA benefits from the ensemble of different scales of patch division and feature re-calibration.

**Figure 7:**
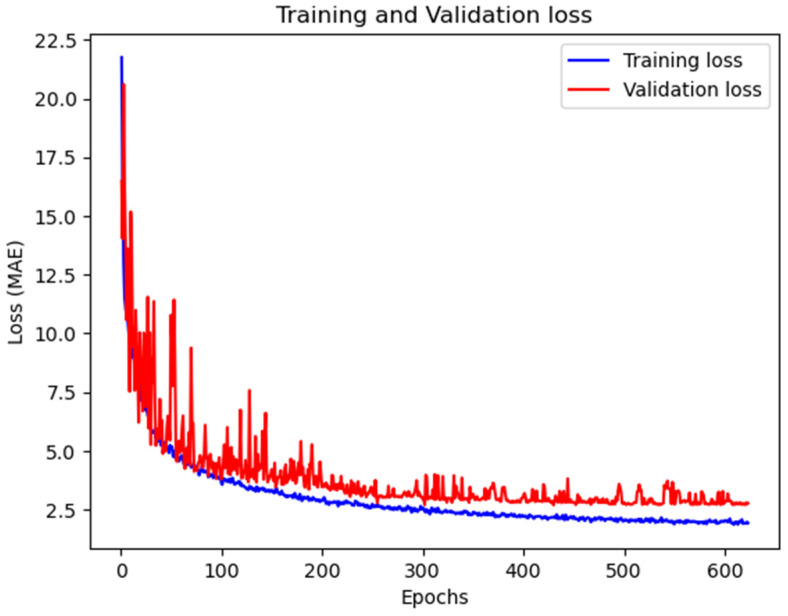
Effect of edge weight coefficient *β* on the performance of brain age prediction.

**Figure 8:**
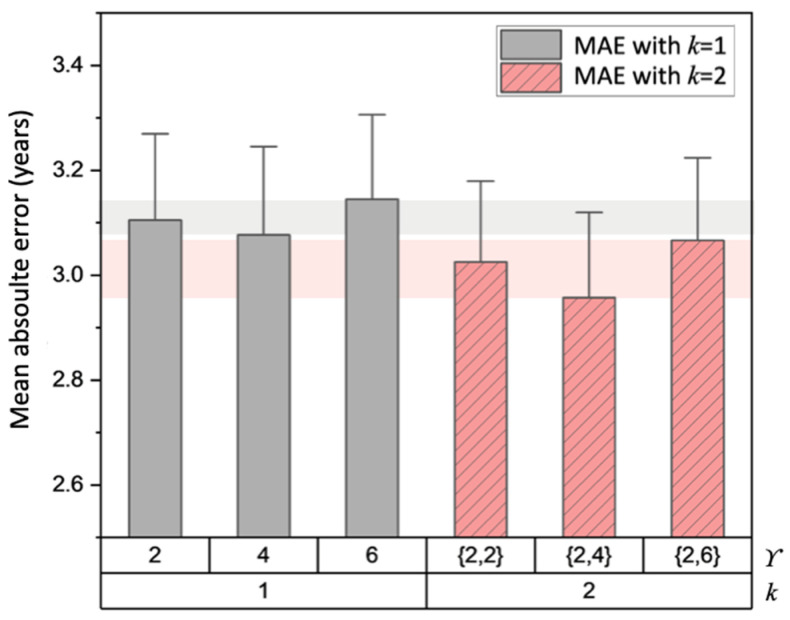
Training and validation loss graph of MGA-sSE-ResNet18. From around 400 epochs onward, the validation loss converges at approximately 2.7 years, which is slightly smaller than the test loss (2.8 years reported in Table 3) and demonstrates effective generalization of our model for unseen data.

The effect of the weight coefficient *β* is shown in Figure 7, where smaller *β* values emphasize small-hop edge matrices (Equation (5)). The value *β* = 0.8 was optimal, resulting in the lowest MAE of 2.822 years. Based on the fine-tuning results, the MGA module’s final hyperparameters were set to *m* = 3, *k* = 2, *ϒ* = {2,4}, and *β* = 0.8, and the MAE loss curve graph corresponding to the condition is shown in Figure 8. These values were used in all subsequent experiments for model comparisons.

### 4.2. Comparison with Multi-Head Self Attention (MSA)

Figure 5 contains the prediction results of MSA-sSE-ResNet18, which is the same as the proposed architecture except that MGA is replaced with MSA. MSA-sSE-ResNet18 yields nearly the same test error as MGA-sSE-ResNet18 with *m* > 5 (the dotted blue line in Figure 5). Those results are because MGA becomes closer to MSA due to the increasing scope of query-to-key attention with increasing m. MSA-sSE-ResNet18 yields a higher MAE (3.216 years) than the proposed MGA-sSE-ResNet18 with an optimal *m* of three and suboptimal *β* of 1.0 (2.957 years). These findings indicate that it is beneficial to consider important embeddings selectively rather than collectively when calculating the self-attention coefficients. It is noteworthy that using MSA instead of MGA with *m* = 3 lowers the training error (MAE = 1.071 vs. 1.539 years) while increasing the test error. This implies that pruning the neighbor nodes for self-attention helps enhance the generalizability of the prediction model.

### 4.3. Comparison with State-of-the-Art Models

Table 3 summarizes the comparison of four general-purpose CNNs (ResNet18, sSE-ResNet18, DenseNet121, and MobileNetV2) and two representative brain age prediction models (the SFCN and TSAN) in terms of the MAE and PCC. All the values were obtained after applying the bias correction to the immediate outputs of the prediction models.

Among the four generic models, sSE-ResNet18 yielded the lowest MAE with improvement over the plain ResNet18 on account of the spatial recalibration of the sSE block. Thus, sSE-ResNet18 was chosen as the backbone network to which the MGA module was connected. DenseNet121 offered the largest MAE presumably due to excessive model parameters, which could not be well optimized by using a limited training dataset. The TSAN revealed the lowest MAE among the six reported models after running both stages. The SFCN underperformed compared with the TSAN, yielding an MAE > 3.0 years, presumably because it was designed for a large training dataset (>10 k).

### 4.4. Ablation Study about MGA

**Figure 9:**
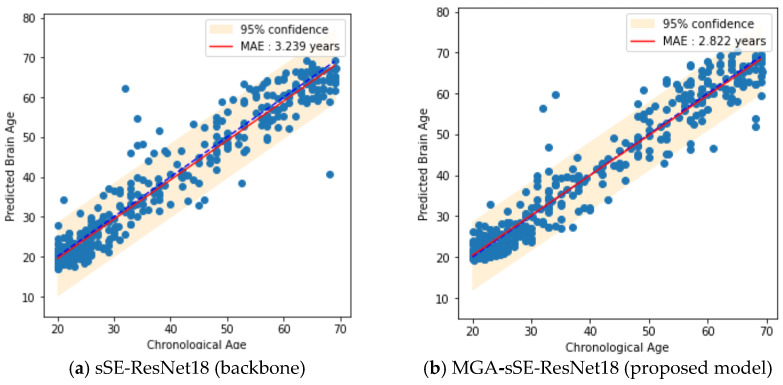
Scatter plots of brain age prediction on test dataset from two different prediction models (backbone and the proposed model). The dotted blue lines represent the ideal prediction, where chronological ages equal predicted ages, and the red lines indicate the linear regressions fitted by model predictions.

Table 3 also shows that MGA’s incorporation into two backbones (ResNet18 and sSE-ResNet18) resulted in improvements over both original networks. Particularly, our final model, MGA-sSE-ResNet18, reduced the MAE by 0.417 years compared to sSE-ResNet18 and achieved the lowest MAE among all the models compared. The improvement made by MGA is reflected in the scatter plots of the prediction outcomes on the test dataset (Figure 9). Compared to sSE-ResNet18, MGA-sSE-ResNet18 reduced the prediction bias and variance, as represented by the red line being closer to the blue line and narrower yellow band, respectively, in Figure 9b. Notably, MSA-sSE-ResNet18 outperformed the compared models except the TSAN. This implies that the strategy of extracting feature patches offers utility for considering the relationship between distant regions. MGA better handles the resultant graph-structured data than MSA by attending to the node neighborhood more optimally. Finally, the additional input of sex information into MGA-sSE-ResNet18 did not reduce the MAE (MAE = 2.859 vs. 2.822 years with and without the sex label).

## 5. Discussion

We developed a multi-hop graph attention module and proposed an MGA-incorporated CNN architecture (MGA-sSE-ResNet18) for predicting brain age based on 3D T1-weighted MR images. MGA represents a novel graph self-attention module that learns the inter-node relationships of graph data formed through patch embeddings of the preceding convolution layer’s output. The capability of considering multi-hop-connected nodes invokes a key MGA feature. This was achieved by deriving an edge matrix accounting for multi-hop transitions across nodes and applying masked attention to the resultant node neighborhood. By applying convolution layers and MGA alternately, the proposed architecture could extract local and global features, the effectiveness of which is verified by demonstrating an improved prediction accuracy over previously reported CNN-based models. The pursuit of both local and global features adheres to ongoing research on self-attention-aided convolutional networks in the natural image domain [59,60]. We expect that, aside from brain age prediction, MGA is applicable to other image-based prediction tasks due to its effective feature extraction and flexible integration into CNNs.

The fine-tuning of the hyperparameter *m* (number of hops) indicates that it is critical to consider the appropriate node neighborhood when implementing masked self-attention in MGA (Figure 5). By performing gate operations on the edge matrix, MGA considers only nodes that are relevant to the target node. The gate operation can be viewed as a simplified version of pruning, used often in tree algorithms [61]. Pruning removes non-significant branches in a tree to mitigate overfitting. Similarly, MGA removes irrelevant nodes after constructing the edge matrix, which improves the model’s generalizability. Another related finding was that MSA integrated with the same backbone (MSA-sSE-ResNet18) yielded nearly the same MAE as the proposed MGA-sSE-ResNet18 with a large *m*. This is consistent with the fact that as *m* increases, the node neighborhood expands, and accordingly, MGA aligns closely with MSA.

ResNet18 combined with an sSE (spatial SE) block served as a backbone network with which the MGA module was integrated. The sSE module spatial-wise recalibrates feature maps by using channel-wise squeeze and spatial-wise excitation. Another earlier reported type of SE module applies the squeeze and excite operations in the opposite dimensions, performing channel-wise re-calibration (channel SE or cSE) [62]. While the initial applications of the cSE and sSE blocks were classification and segmentation, respectively, sSE was chosen based on our experimental finding that sSE-ResNet18 outperformed cSE-ResNet18 as well as plain ResNet18 in brain age prediction. Note that, accordingly, MGA-sSE-ResNet18 produced a lower MAE than MGA-ResNet18 as reported in Table 3.

The proposed method has the advantages of the capability to learn both local and global image features and flexibility in combination with existing convolutional neural networks. Nevertheless, it also involves some limitations, summarized as follows: (1) Generally present in graph algorithms, MGA requires expansive memory for training, particularly when the network is configured for 3D input. With a total GPU memory of 96 GB available, we could implement only up to two branches of patch embedding (*k* = 2) without encountering memory errors. (2) The size of the labeled dataset in this study was limited (2788) compared to other recent studies (e.g., 14,503 subjects in [1]). This may have limited the performance of the proposed and compared networks, some of which were optimized with larger datasets. (3) The lack of validation in diseased patients poses another limitation. This study focused on improving the accuracy of brain age prediction for healthy subjects, which serves as an essential tool for estimating deviations from normal aging. The value of brain age estimation should, however, be assessed in a cohort of patients with degenerative neurological diseases.

## 6. Conclusions

We proposed a multi-hop graph attention module (MGA) integrated into a 3D CNN for brain age prediction based on 3D brain MR images. MGA successfully learns the relationships between the multi-hop-connected nodes of a graph set formed through the patch split and embedding of feature tensors. By interleaving MGA with conventional convolutional layers, the proposed architecture can extract the local and global features of anatomical brain images and enhance the representational capacity. This reflects the improved accuracy of brain age prediction compared to previously reported convolutional models.

## Figures and Tables

**Table 1:** Detailed settings for MGA-sSE-ResNet18. Building blocks are stacked in table cells, where the two cells for sSE and MGA modules are in the same row indicating their parallel connection. The detailed structure of MGA appears in Figure 1 while its hyperparameter values of *m* = 3, *k* = 2, *ϒ* = {2, 4}, and *β* = 0.8 are determined based on the fine-tuning results.

Output Size	MGA-sSE-ResNet18		
101 × 101 × 121	7 × 7 × 7, 64, stride 2		
50 × 50 × 60	3 × 3 × 3, max pool, stride 2		
	Conv, 3 × 3 × 3, 64 Conv, 3 × 3 × 3, 64		×2
	sSE: Conv, 1 × 1 × 1, 64 Sigmoid	MGA: *m* = 3, *k* = 2, *ϒ* = {2, 4}, *β* = 0.8	
25 × 25 × 30	Conv, 3 × 3 × 3, 128 Conv, 3 × 3 × 3, 128		×2
	sSE: Conv, 1 × 1 × 1, 128 Sigmoid	MGA: *m* = 3, *k* = 2, *ϒ* = {2, 4}, *β* = 0.8	
12 × 12 × 15	Conv, 3 × 3 × 3, 256 Conv, 3 × 3 × 3, 256		×2
	sSE: Conv, 1 × 1 × 1, 256 Sigmoid	MGA: *m* = 3, *k* = 2, *ϒ* = {2, 4}, *β* = 0.8	
6 × 6 × 7	Conv, 3 × 3 × 3, 512 Conv, 3 × 3 × 3, 512		×2
	sSE: Conv, 1 × 1 × 1, 512 Sigmoid	MGA: *m* = 3, *k* = 2, *ϒ* = {2, 4}, *β* = 0.8	
1 × 1 × 1	Global average pool, 1-d fc, softmax		

**Table 2:** Demographic information of the brain MRI dataset for brain age prediction.

	N_samples_	Female	Male	Mean Age	Min Age	Max Age
OpenNeuro	542	323	219	26.71	20	69
COBRE	71	22	49	36.45	20	65
Open fMRI	353	170	183	35.85	20	69
INDI	696	398	298	50.23	30	69
IXI	123	61	62	50.54	30.89	69.55
FCP1000	835	477	358	27.50	20	69
XNAT	168	0	168	63.46	42	69

**Table 3:** Comparisons with other models including four generic CNNs, two brain age prediction models, and three MGA-CNN combinations.

Model	MAE	PCC
ResNet18	3.249	0.948
sSE-ResNet18	3.239	0.956
DenseNet121	3.340	0.961
MobileNetV2	3.295	0.950
SFCN	3.233	0.949
TSAN	2.892	0.956
MSA-sSE-ResNet18	3.216	0.960
MGA-sSE-ResNet18	2.822	0.968
MGA-ResNet18	3.065	0.955
MGA-sSE-ResNet18 (with sex label)	2.859	0.960

## Data Availability

Publicly available datasets were used in this study.

## References

[1] Cole J.H., Franke K. (2017). Predicting age using neuroimaging: Innovative brain ageing biomarkers. Trends Neurosci..

[2] Franke K., Gaser C. (2019). Ten years of BrainAGE as a neuroimaging biomarker of brain aging: What insights have we gained?. Front. Neurol..

[3] Cole J.H., Ritchie S.J., Bastin M.E., Hernández V., Muñoz Maniega S., Royle N., Corley J., Pattie A., Harris S.E., Zhang Q. (2017). Brain age predicts mortality. Mol. Psychiatry.

[4] Lewis J.D., Evans A.C., Tohka J., Brain Development Cooperative Group (2018). T1 white/gray contrast as a predictor of chronological age, and an index of cognitive performance. Neuroimage.

[5] Jonsson B.A., Bjornsdottir G., Thorgeirsson T.E., Ellingsen L.M., Walters G.B., Gudbjartsson D.F., Stefansson H., Stefansson K., Ulfarsson M.O. (2019). Brain age prediction using deep learning uncovers associated sequence variants. Nat. Commun..

[6] Chen C.-L., Hsu Y.-C., Yang L.-Y., Tung Y.-H., Luo W.-B., Liu C.-M., Hwang T.-J., Hwu H.-G., Tseng W.-Y.I. (2020). Generalization of diffusion magnetic resonance imaging–based brain age prediction model through transfer learning. NeuroImage.

[7] Cole J.H., Raffel J., Friede T., Eshaghi A., Brownlee W.J., Chard D., De Stefano N., Enzinger C., Pirpamer L., Filippi M. (2020). Longitudinal assessment of multiple sclerosis with the brain-age paradigm. Ann. Neurol..

[8] Peng H., Gong W., Beckmann C.F., Vedaldi A., Smith S.M. (2020). Accurate brain age prediction with lightweight deep neural networks. Med. Image Anal..

[9] Cole J.H., Leech R., Sharp D.J., Alzheimer’s Disease Neuroimaging Initiative (2015). Prediction of brain age suggests accelerated atrophy after traumatic brain injury. Ann. Neurol..

[10] Du L., Roy S., Wang P., Li Z., Qiu X., Zhang Y., Yuan J., Guo B. (2024). Unveiling the Future: Advancements in MRI Imaging for Neurodegenerative Disorders. Ageing Res. Rev..

[11] Wang J., Knol M.J., Tiulpin A., Dubost F., de Bruijne M., Vernooij M.W., Adams H.H.H., Ikram M.A., Niessen W.J., Roshchupkin G.V. (2019). Gray matter age prediction as a biomarker for risk of dementia. Proc. Natl. Acad. Sci. USA.

[12] Biondo F., Jewell A., Pritchard M., Mueller C., Steves C.J., Cole J. (2020). Brain-age predicts subsequent dementia in memory clinic patients. medRxiv.

[13] Gaser C., Franke K., Klöppel S., Koutsouleris N., Sauer H., Alzheimer’s Disease Neuroimaging Initiative (2013). BrainAGE in mild cognitive impaired patients: Predicting the conversion to Alzheimer’s disease. PLoS ONE.

[14] Beheshti I., Maikusa N., Matsuda H. (2018). The association between “brain-age score”(BAS) and traditional neuropsychological screening tools in Alzheimer’s disease. Brain Behav..

[15] Koutsouleris N., Davatzikos C., Borgwardt S., Gaser C., Bottlender R., Frodl T., Falkai P., Riecher-Rössler A., Möller H.-J., Reiser M. (2013). Accelerated brain aging in schizophrenia and beyond: A neuroanatomical marker of psychiatric disorders. Schizophr. Bull..

[16] Koutsouleris N., Meisenzahl E.M., Borgwardt S., Riecher-Rössler A., Frodl T., Kambeitz J., Köhler Y., Falkai P., Möller H.-J., Reiser M. (2015). Individualized differential diagnosis of schizophrenia and mood disorders using neuroanatomical biomarkers. Brain.

[17] Kuchinad A., Schweinhardt P., Seminowicz D.A., Wood P.B., Chizh B.A., Bushnell M.C. (2007). Accelerated brain gray matter loss in fibromyalgia patients: Premature aging of the brain?. J. Neurosci..

[18] Liem F., Varoquaux G., Kynast J., Beyer F., Masouleh S.K., Huntenburg J.M., Lampe L., Rahim M., Abraham A., Craddock R.C. (2016). Predicting brain-age from multimodal imaging data captures cognitive impairment. NeuroImage.

[19] Chung Y., Addington J., Bearden C.E., Cadenhead K., Cornblatt B., Mathalon D.H., McGlashan T., Perkins D., Seidman L.J., Tsuang M. (2018). Use of machine learning to determine deviance in neuroanatomical maturity associated with future psychosis in youths at clinically high risk. JAMA Psychiatry.

[20] Schnack H.G., van Haren N.E., Nieuwenhuis M., Pol H.E.H., Cahn W., Kahn R.S. (2016). Accelerated brain aging in schizophrenia: A longitudinal pattern recognition study. Am. J. Psychiatry.

[21] Guan S., Jiang R., Meng C., Biswal B. (2023). Brain age prediction across the human lifespan using multimodal MRI data. GeroScience.

[22] LeCun Y., Bengio Y., Hinton G. (2015). Deep learning. Nature.

[23] Cole J.H., Poudel R.P., Tsagkrasoulis D., Caan M.W., Steves C., Spector T.D., Montana G. (2017). Predicting brain age with deep learning from raw imaging data results in a reliable and heritable biomarker. NeuroImage.

[24] Jiang H., Lu N., Chen K., Yao L., Li K., Zhang J., Guo X. (2020). Predicting brain age of healthy adults based on structural MRI parcellation using convolutional neural networks. Front. Neurol..

[25] Jiang H., Guo J., Du H., Xu J., Qiu B. (2019). Transfer learning on T1-weighted images for brain age estimation. Math. Biosci. Eng..

[26] Lam P., Zhu A.H., Gari I.B., Jahanshad N., Thompson P.M. (2020). 3D Grid-Attention Networks for Interpretable Age and Alzheimer’s Disease Prediction from Structural MRI. arXiv.

[27] Cheng J., Liu Z., Guan H., Wu Z., Zhu H., Jiang J., Wen W., Tao D., Liu T. (2021). Brain age estimation from MRI using cascade networks with ranking loss. IEEE Trans. Med. Imaging.

[28] He S., Pereira D., Perez J.D., Gollub R.L., Murphy S.N., Prabhu S., Ou Y. (2021). Multi-channel attention-fusion neural network for brain age estimation: Accuracy, generality, and interpretation with 16,705 healthy MRIs across lifespan. Med. Image Anal..

[29] Zhang Y., Xie R., Beheshti I., Liu X., Zheng G., Wang Y., Zhang Z., Zheng W., Yao Z., Hu B. (2024). Improving brain age prediction with anatomical feature attention-enhanced 3D-CNN. Comput. Biol. Med..

[30] Sporns O. (2007). Brain connectivity. Scholarpedia.

[31] Jun E., Jeong S., Heo D.W., Suk H.I. (2021). Medical transformer: Universal brain encoder for 3D MRI analysis. arXiv.

[32] He S., Grant P.E., Ou Y. (2021). Global-Local transformer for brain age estimation. IEEE Trans. Med. Imaging.

[33] Kawahara J., Brown C.J., Miller S.P., Booth B.G., Chau V., Grunau R.E., Zwicker J.G., Hamarneh G. (2017). BrainNetCNN: Convolutional neural networks for brain networks; towards predicting neurodevelopment. NeuroImage.

[34] Liu M., Kim S., Duffy B., Yuan S., Cole J.H., Toga A.W., Kim H. (2021). Brain age predicted using graph convolutional neural network explains developmental trajectory in preterm neonates. bioRxiv.

[35] Cai H., Gao Y., Liu M. (2022). Graph transformer geometric learning of brain networks using multimodal MR images for brain age estimation. IEEE Trans. Med. Imaging.

[36] Roy A.G., Navab N., Wachinger C. (2018). Recalibrating fully convolutional networks with spatial and channel “squeeze and excitation” blocks. IEEE Trans. Med. Imaging.

[37] Bao L., Ma B., Chang H., Chen X. Masked graph attention network for person re-identification. Proceedings of the IEEE/CVF Conference on Computer Vision and Pattern Recognition Workshops.

[38] Dong Y., Liu Q., Du B., Zhang L. (2022). Weighted feature fusion of convolutional neural network and graph attention network for hyperspectral image classification. IEEE Trans. Image Process..

[39] Veličković P., Cucurull G., Casanova A., Romero A., Lio P., Bengio Y. (2017). Graph attention networks. arXiv.

[40] Liao R., Li Y., Song Y., Wang S., Hamilton W., Duvenaud D.K., Zemel R. (2019). Efficient graph generation with graph recurrent attention networks. Adv. Neural Inf. Process. Syst..

[41] Xiong Z., Wang D., Liu X., Zhong F., Wan X., Li X., Li Z., Luo X., Chen K., Jiang H. (2019). Pushing the boundaries of molecular representation for drug discovery with the graph attention mechanism. J. Med. Chem..

[42] Yang Y., Wang X., Song M., Yuan J., Tao D. (2021). Spagan: Shortest path graph attention network. arXiv.

[43] He K., Zhang X., Ren S., Sun J. Deep residual learning for image recognition. Proceedings of the IEEE Conference on Computer Vision and Pattern Recognition (CVPR).

[44] Liao L., Zhang X., Zhao F., Lou J., Wang L., Xu X., Zhang H., Li G. Multi-branch deformable convolutional neural network with label distribution learning for fetal brain age prediction. Proceedings of the 2020 IEEE 17th International Symposium on Biomedical Imaging (ISBI).

[45] Gorgolewski K., Esteban O., Schaefer G., Wandell B., Poldrack R. (2017). OpenNeuro—A Free Online Platform for Sharing and Analysis of Neuroimaging Data.

[46] Mayer A.R., Ruhl D., Merideth F., Ling J., Hanlon F.M., Bustillo J., Cañive J. (2012). Functional imaging of the hemodynamic sensory gating response in schizophrenia. Hum. Brain Mapp..

[47] Poldrack R.A., Barch D.M., Mitchell J.P., Wager T.D., Wagner A.D., Devlin J.T., Cumba C., Koyejo O., Milham M.P. (2013). Toward open sharing of task-based fMRI data: The OpenfMRI project. Front. Neurosci..

[48] Mennes M., Biswal B.B., Castellanos F.X., Milham M.P. (2013). Making data sharing work: The FCP/INDI experience. NeuroImage.

[49] Simonovsky M., Gutiérrez-Becker B., Mateus D., Navab N., Komodakis N. A deep metric for multimodal registration. Proceedings of the International Conference on Medical Image Computing and Computer-Assisted Intervention.

[50] Biswal B.B., Mennes M., Zuo X.-N., Gohel S., Kelly C., Smith S.M., Beckmann C.F., Adelstein J.S., Buckner R.L., Colcombe S. (2010). Toward discovery science of human brain function. Proc. Natl. Acad. Sci. USA.

[51] Herrick R., Horton W., Olsen T., McKay M., Archie K.A., Marcus D.S. (2016). XNAT Central: Open sourcing imaging research data. NeuroImage.

[52] Song S., Zheng Y., He Y. (2017). A review of methods for bias correction in medical images. Biomed. Eng. Rev..

[53] Larsson G., Maire M., Shakhnarovich G. (2016). Fractalnet: Ultra-deep neural networks without residuals. arXiv.

[54] Smith S.M., Vidaurre D., Alfaro-Almagro F., Nichols T.E., Miller K.L. (2019). Estimation of brain age delta from brain imaging. NeuroImage.

[55] Vaswani A., Shazeer N., Parmar N., Uszkoreit J., Jones L., Gomez A.N., Kaiser Ł., Polosukhin I. (2017). Attention is all you need. Adv. Neural Inf. Process. Syst..

[56] Dosovitskiy A., Beyer L., Kolesnikov A., Weissenborn D., Zhai X., Unterthiner T., Houlsby N. (2020). An image is worth 16 × 16 words: Transformers for image recognition at scale. arXiv.

[57] Huang G., Liu Z., Van Der Maaten L., Weinberger K.Q. Densely connected convolutional networks. Proceedings of the IEEE Conference on Computer Vision and Pattern Recognition.

[58] Howard A.G., Zhu M., Chen B., Kalenichenko D., Wang W., Weyand T., Adam H. (2017). Mobilenets: Efficient convolutional neural networks for mobile vision applications. arXiv.

[59] Li Y., Zeng J., Shan S., Chen X. (2018). Occlusion aware facial expression recognition using CNN with attention mechanism. IEEE Trans. Image Process..

[60] Guo Y., Ji J., Lu X., Huo H., Fang T., Li D. (2019). Global-local attention network for aerial scene classification. IEEE Access.

[61] Reed R. (1993). Pruning algorithms—A survey. IEEE Trans. Neural Netw..

[62] Hu J., Shen L., Sun G. Squeeze-and-excitation networks. Proceedings of the IEEE Conference on Computer Vision and Pattern Recognition.

